# Obtaining anthocyanin from jambolan fruit: Kinetics, extraction rate, and prediction of process time for different agitation frequencies

**DOI:** 10.1002/fsn3.730

**Published:** 2018-07-20

**Authors:** Wilton Pereira da Silva, Jarderlany Sousa Nunes, Josivanda Palmeira Gomes, Cleide Maria Diniz Pereira da Silva e Silva

**Affiliations:** ^1^ Federal University of Campina Grande Campina Grande Brazil

**Keywords:** anthocyanin, extraction kinetics, extraction rate, process time determination

## Abstract

In the literature, in general, the kinetics of bioactive compounds extraction from a solid–liquid system is described by diffusion models and by the Peleg model. In this study, four experiments on the kinetics of anthocyanin extraction from jambolan fruit, at different agitation frequencies of the medium (0, 50, 100, and 150 rpm), are described by various empirical models with up to two fit parameters. According to the statistical indicators, the best model to describe the kinetic processes was Page's, which was also used to determine the extraction rates (all decreasing) and estimate the process times for each agitation frequency. The extraction time for the 150 rpm frequency is approximately six times shorter than that for 0 rpm. Thus, the 150 rpm frequency can be recommended for industrial applications, as the process time is an important variable in the production cost.

## 1 INTRODUCTION

Food dyes are additives added to foods to intensify their color, making their appearance more pleasing to the consumer's eye. These products are important because they can increase the acceptability of food products. Due to the large‐scale utilization of substances used as dyes in foods and beverages, their control became necessary because of the concern about possible negative effects on human health. In the last years, the food safety of synthetic pigments has been questioned, which has led to a reduction in the number of this type of product allowed by regulatory norms of various countries. Consequently, the interest in natural dyes increased substantially, especially due to the apparent absence of toxicity (Chethana, Chetan, & Raghavarao, 2007; Giusti & Wrolstad, 2003).

In general, natural pigments are bioactive products, which, besides performing the function of enhancing the color of foods and beverages, in general promote health and well‐being. In this context, anthocyanins stand out, which are glycosides of polyhydroxy and polymethoxy derivatives of 2‐phenylbenzopyrylium salts (flavylium) that define the colors of various fruits, vegetables, and flowers. Anthocyanins are obtained from a wide range of plant species, and some of them are already used by the industry as sources of extraction. Due to the polar nature, anthocyanins are soluble in water or in polar organic solvents, which facilitates their incorporation in aqueous food systems (Dyrby, Wesergaard, & Stapelfeldt, 2001; Hasler, 2000).

Natural presence of anthocyanins in an agricultural product is usually associated with colorful, attractive fruits such as grapes, strawberries, raspberries, pomegranates, mangoes, figs, among others. One of these fruits is jambolan (*Syzygium cumini* (L.)), which exhibits an intense purple color, due to the high content of anthocyanin compounds, equally present in fruits such as grape (*Vitis* sp.), bilberry (*Vaccinium myrtillus*), and “jabuticaba” (*Myrciaria cauliflora*). It is interesting to note that one of the special features of these compounds is their high solubility in aqueous mixtures. On the other hand, anthocyanin contents found in jambolan fruits are similar to those detected in blueberries, which are already considered as a nutraceutical commodity of high commercial importance. The high antioxidant activity in jambolan extract (along with great dyeing potential, with the desirable attributes of solubility and stability) can stimulate the inclusion of this extract as natural additive in both foods and pharmaceutical formulations (Francis, 1989; Liu et al., 2010; Veigas, Narayan, Laxman, & Neelwarne, 2007).

Bioactive compounds such as anthocyanins are usually obtained by solid–liquid extraction. This process is influenced by the chemical nature, extraction method used, particle size, immersion time in liquid medium, and processing conditions, as well as the presence of interfering substances. Alcoholic solvents and the use of acids favor the extraction process, because they facilitate solvent penetration in the tissues of fruits and vegetables, besides increasing the stability of the extracts for hindering the appearance of fungi that degrade this type of product. Acid medium also causes anthocyanins, in particular, to be found predominantly in the form of flavylium cation, which exhibits red color in aqueous solution (Revilla, Ryan, & Martin‐Ortega, 1998).

To describe and optimize the extraction process, authors normally use the Fick's law (Bonfigli, Godoy, Reinheimer, & Scenna, 2017; Bucic‐Kojic, Planinic, Tomas, Bilic, & Valic, 2007; Cacace & Mazza, 2003; Cissé et al., 2012; Espinoza‐Perez, Vargas, Robles‐Olvera, Rodríguez‐Jimenes, & Garcia‐ Alvarado, 2007; Garcia‐Perez, García‐Alvarado, Carce, & Mulet, 2010; Tao, Zhang, & Sun, 2014). However, dye extraction kinetics is also described by empirical equations (D'Alessandro, Dimitrov, Vauchel, & Nikov, 2013; Lin, Xia, & Liu, 2017; Pan, Qu, Mab, Atungulu, & McHugh, 2011), generally using the Peleg equation, which can also be interpreted as a second‐order rate model (Pan et al., 2011). In the literature consulted, no studies were found using other empirical models to describe this type of process. In this context, the objectives of this study are defined below.

This paper aimed to: (a) conduct experiments involving anthocyanin extraction from jambolan fruits using different stirring frequencies of the medium; (b) propose several empirical models to describe the extraction process, choosing the one with best statistical indicators; and (c) determine the process time and propose the best experimental arrangement, among the analyzed ones, to be considered by the industry.

## 2 MATERIAL AND METHODS

### 2.1 Experiments

Ripe jambolan (*Syzygium cumini*) fruits were collected from a farm located in the municipality of Campina Grande, PB, Brazil. The fruits were washed to remove impurities and rinsed in running water. Sanitization consisted in the immersion of the fruits in 200 mg/L sodium hypochlorite solution (free chlorine) for 20 min. After that, they were immersed in potable water for rinsing. The fruits were pulped in an industrial pulping machine to not only remove the seeds, but also grind the pulp. The product was dried at 40°C in a forced‐air oven for 48 hours, and the average radius of the obtained granules was about 0.92 mm.

Solid‐to‐solvent ratio was chosen as 1:20, based on works of Cissé et al. (2012) and D'Alessandro et al. (2013). Solvent was composed of 70% ethyl alcohol and hydrochloric acid at pH 3.0 (85:15 ratio), and the extraction temperature was fixed at 35°C. In order to extract anthocyanins, a refrigerated orbital incubator of the brand Tecnal^®^ (Brazil), model TE‐421, was used. This equipment allows to control the temperature between 0 and 60 °C, and to agitate a horizontal metal plate with an Erlenmeyer at frequencies between 30 and 250 rpm. The kinetic study was conducted using four different stirring frequencies (0, 50, 100, and 150 rpm). At specific time instants (0, 2, 4, 6, 8, 10, 15, 20, 30,…, 120, and 130 min), anthocyanin concentration was determined through the method described by Francis (1982), with readings in UV–Vis spectrophotometer at 535 nm wavelength. Blank was established only for the ethanol‐HCl solution (1.5 N).

### 2.2 Empirical models

To describe the anthocyanin extraction kinetics through empirical models, the following assumptions were established: (a) The number of fitting parameters of the empirical equations should be only one or two; (b) A mathematical expression for the extraction rate as a function of the time should be obtained from the empirical equation; (c) A mathematical expression for the extraction time as a function of the concentration must be obtained from the empirical equation. Thus, the dimensionless concentration X* at time *t* should be given by:

(1) $$X^{*}\left(t\right)=\frac{X\left(t\right)-X_{\mathrm{eq}}}{X_{i}-X_{\mathrm{eq}}}=f\left(t,a,b\right)$$

in which *X(t)* is the anthocyanin concentration at instant *t*,* X*_*eq*_ is the equilibrium concentration, *X*_*i*_ is the initial concentration, *a* and *b* are fitting parameters. From Equation (1), the following equation can be written for the concentration at instant *t*:

(2) $$X\left(t\right)=X_{\mathrm{eq}}+\left(X_{i}-X_{\mathrm{eq}}\right)f\left(t,a,b\right).$$

As the loss of anthocyanins from the granules to the medium is initially zero, Equation (2) can be rewritten in the following way:

(3) $$X\left(t\right)=X_{\mathrm{eq}}\left[1-f\left(t,a,b\right)\right].$$

Table 1 presents five empirical functions *f(t,a,b)* that obey the earlier assumptions and, at first, they can be used to describe the process.

**Table 1:** Empirical functions to describe anthocyanin loss to the liquid medium

Model	Name	Empirical functions X* = *f(t,a,b)*	Reference
1	Lewis	*e* ^−*at*^	Kaleta and Górnicki (2010)
2	Henderson and Pabis	*ae* ^−*bt*^	Diamante, Ihns, Savage, and Vanhanen (2010)
3	Peleg	1−*t/*(*a* + *bt*)	Mercali, Tessaro, Norena, and Marczak (2010)
4	Page	$e^{-at^{b}}$	Diamante et al. (2010)
5	Silva et alii	$e^{-at-b\sqrt{t}}$	Silva et al. (2013)

The functions given in Table 1 can substitute the generic function *f(t,a,b)* given in Equation (3), in order to describe the anthocyanin extraction process by the liquid medium.

The extraction rate at instant *t* is calculated by determining the derivative of Equation (3) with respect to time:

(4) $$\frac{\mathrm{dX}}{\mathrm{dt}}=-X_{\mathrm{eq}}\frac{\mathrm{df}}{\mathrm{dt}}.$$

The derivative of each empirical function given in Table 1 with respect to time, as well as the extraction time for a given dimensionless concentration X*, is shown in Table 2.

**Table 2:** Derivative of the empirical functions and extraction time

Model	*df/dt*	Extraction time
1	−*ae* ^*−at*^	*t* = −lnX*/*a*
2	−*abe* ^−*bt*^	*t* = −ln(X*/*a*)/*b*
3	−*a*/(*a* + *bt*)^2^	*t* = *a*(1−X*)/(1−*b* + b X*)
4	$-abt^{b-1}e^{-at^{b}}$	$t={\left(-\text{ln}X^{*}/a\right)}^{1/b}$
5	$-\left(a+bt^{-1/2}/2\right)e^{-at-b\sqrt{t}}$	$t={\left[\left(-b\pm \sqrt{b^{2}-4a\text{ln}X^{*}}\right)/\left(2a\right)\right]}^{2}$

## 3 RESULTS AND DISCUSSION

### 3.1 Experimental data

Concentrations of anthocyanins extracted from the granules by the liquid medium over time were obtained at 35°C, with the following agitation frequencies: 0, 50, 100, and 150 rpm. Experimental data of extraction kinetics at 150 rpm frequency allowed to estimate the equilibrium concentration, based on the arithmetic mean of the values of the last concentrations obtained, which resulted in *X*_*eq*_ = 13.1 mg/100 g.

### 3.2 Empirical models: Extraction kinetics

Equation (3), written for each empirical model presented in Table 1, was fitted to the experimental datasets, using nonlinear regression through LAB Fit Curve Fitting Software (Da Silva et al., 2004). The results, given in Table 3, were evaluated through the statistical indicators chi‐square, χ^2^, and determination coefficient, *R* ^*2*^ (Bevington & Robinson, 1992; Da Silva, Mata, Silva, Guedes, & Lima, 2008; Taylor, 1997).

**Table 3:** Results obtained for the models

Model	Frequency (rpm)	Parameters of *a*	model *b*	*R* ^2^	χ^2^
Lewis	0	2.843 × 10^−2^	–	0.9941	4.7848
	50	4.6611 × 10^−2^	–	0.9869	5.2828
	100	8.603 × 10^−2^	–	0.9865	8.3106
	150	1.882 × 10^−1^	–	0.9864	3.6293
Henderson and Pabis	0	0.9465	2.610 × 10^−2^	0.9921	2.9891
	50	1.001	4.666 × 10^−2^	0.9869	5.2827
	100	0.9807	8.338 × 10^−2^	0.9851	2.2113
	150	0.9445	1.760 × 10^−1^	0.9868	2.9932
Peleg	0	24.88	0.8592	0.9960	1.3431
	50	15.42	0.8832	0.9838	5.3691
	100	7.520	0.9534	0.9894	3.3609
	150	2.978	0.9535^2^	0.9914	1.9080
Page	0	5.171 × 10^−2^	0.8319	0.9967	1.1283
	50	6.161 × 10^−2^	0.9027	0.9861	4.6047
	100	1.486 × 10^−1^	0.7538	0.9840	5.4106
	150	3.047 × 10^−1^	0.7336	0.9961	0.8868
Silva et alii	0	1.989 × 10^−2^	4.722 × 10^−2^	0.9960	1.3854
	50	4.118 × 10^−2^	2.134 × 10^−2^	0.9902	5.0994
	100	4.803 × 10^−2^	1.110 × 10^−1^	0.9818	6.8611
	150	8.739 × 10^−2^	2.430 × 10^−1^	0.9968	0.7265

**Figure 1:**
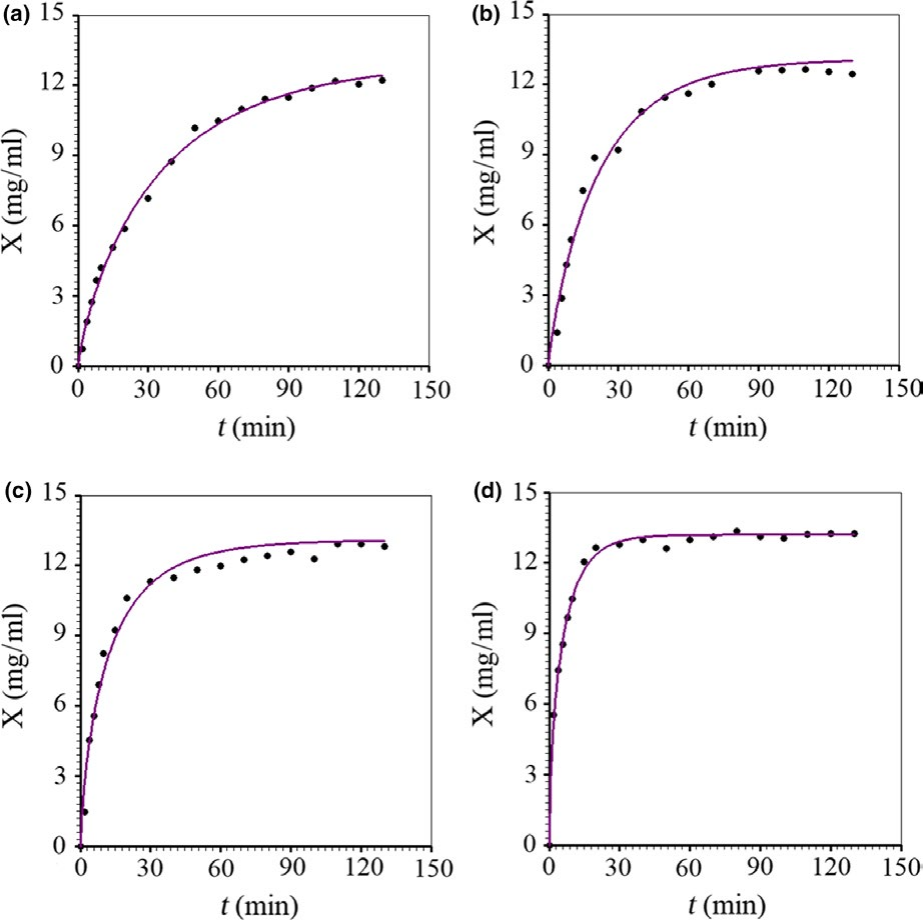
Kinetics of anthocyanin loss by the granules to the liquid medium at 35°C, described by the Page model, with agitation frequency of the medium of: (a) 0 rpm; (b) 50 rpm; (c) 100 rpm; (d) 150 rpm

According to the statistical indicators in Table 3, in general all empirical models proposed represent the studied extraction process reasonably well. It is worth highlighting that the Peleg model can be interpreted as an equation that results from the second‐order concentration rate law, which allows to give a physical meaning to the parameters obtained by curve fitting (Pan et al., 2011; Tao et al., 2014). Despite that, this model was just the second best for the frequency 0 and 100 rpm. On the other hand, although the equation of Silva, Silva, Sousa, and Farias (2013) was the best model for the highest agitation frequency, 150 rpm, the Page model was the best one, or the second best, for three of the four agitation frequencies of the extraction medium. Thus, the Page model was chosen to represent the process, as presented in Figure 1.

Figure 1 shows that, for the frequencies of 0 and 50 rpm (and possibly for 100 rpm), the extraction kinetics did not yet reach the equilibrium concentration. This helps explain why the equilibrium concentration was determined using the arithmetic mean of the last values obtained for the 150 rpm frequency.

As the Page model was chosen to represent the process, the rate of anthocyanin extraction from the granules by the liquid medium, given by Equation (4), can be determined by deriving the Page equation with respect to time (Table 2), which in the present case leads to the following expression (Silva, Silva, Gama, & Gomes, 2014):

(5) $$dX/dt=X_{\mathrm{eq}}abt^{b-1}e^{-at^{b}}.$$

**Figure 2:**
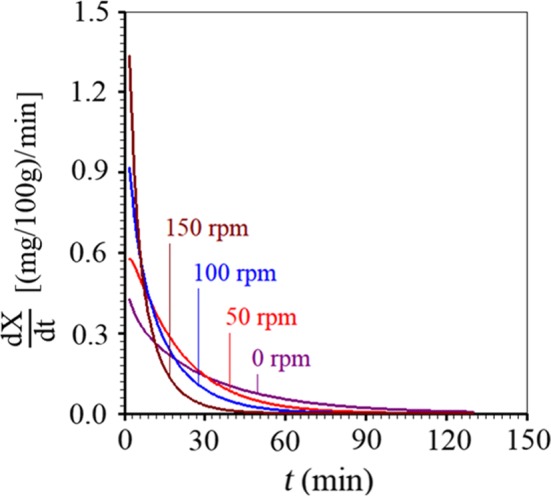
Rate of anthocyanin extraction from the granules by the liquid medium for all experimental conditions

Thus, for the four experiments, the extraction rates are given as shown in Figure 2.

In Figure 2, it is possible to note that, at the beginning of the process, the higher the agitation frequency of the medium, the higher the extraction rate. In addition, along the entire time, for all frequencies, the process occurs at a decreasing rate from the initial instants until the end, that is, the moment in which the equilibrium starts. From this moment on, the extraction period ends and *dX/dt* obviously assumes the zero value.

**Figure 3:**
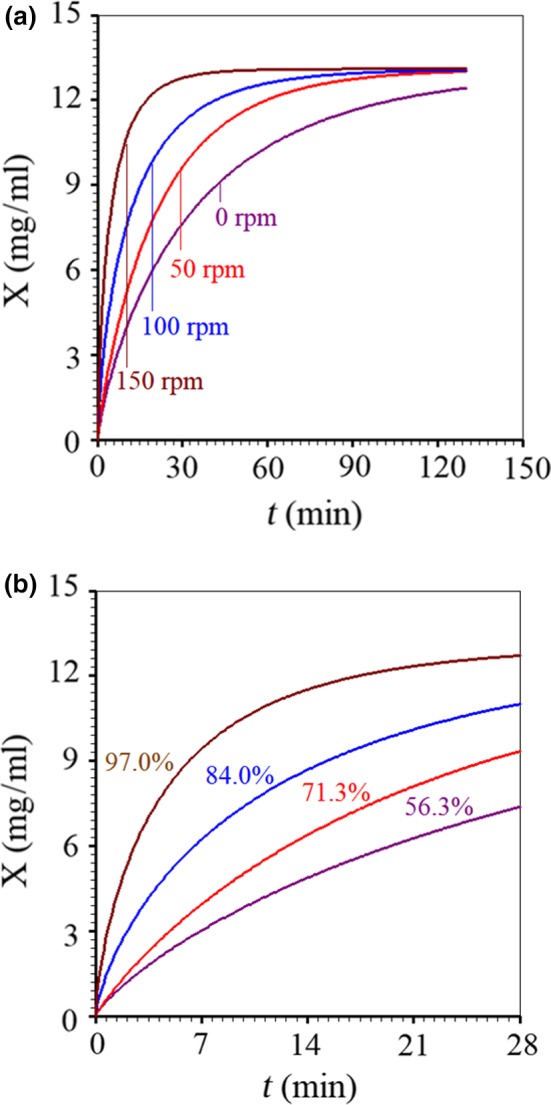
Superposition of anthocyanin extraction kinetics for agitation frequencies of 0, 50, 100, and 150 rpm until the instants: (a) 130 min and (b) 28 min, indicating the concentration *X*(28 min) as percentage of the equilibrium concentration *X*_*eq*_

The process time to reach a given concentration of anthocyanin extraction, for a given agitation frequency, could be estimated by the graphs shown in Figure 1. However, it is more accurate to use the definition of inverse function applied to Equation (1), in which the function *f(t,a,b)* was chosen as the Page equation. In this case, the expression for extraction time using the Page equation is given in Table 2 by model 4. As an example, the time for anthocyanin concentration to be 97% of the equilibrium value (*X(t)* = 0.97*X*_*eq*_ and, therefore, by Equation (1), X* = 0.03) can be estimated using the following expression:

(6) $$t={\left(-\text{ln}\;0.03/a\right)}^{1/b},$$

where *a* and *b* are Page equation parameters obtained for each agitation frequency, as demonstrated in Table 3. Thus, for the frequencies of 0, 50, 100, and 150 rpm, the times required for anthocyanin concentration to reach 0.97*X*_*eq*_ are approximately 159, 88, 66, and 28 min, respectively. These results make even more evident the effect of agitation frequency on the quickness of the anthocyanin extraction process. The superposition of the simulations of extraction kinetics (using the Page model), for various agitation frequencies, can be presented in Figure 3a. On the other hand, Figure 3(b) presents the superposition of the extraction kinetics only for the first 28 min of process.

Based on Figure 3a, indeed, the extraction kinetics with 150 rpm agitation frequency better defines the equilibrium concentration, which justifies its determination through the mean of the last values obtained for this frequency. In contrast, Figure 3b provides an important information that may be considered as useful by the industry: at *t *=* *28 min, 97.0% of the equilibrium concentration of anthocyanins has already been extracted, at stirring frequency of 150 rpm. At this same instant, only 84.0%, 71.3%, and 56.3% of the equilibrium concentrations have been extracted at 100, 50, and 0 rpm, respectively.

## 4 CONCLUSIONS

The obtained extract has a great antioxidant potential, being able to be used in the industries of foods, cosmetics, and medicines, in place of similar artificial products. In this context, as result of this study, it was possible to conclude that: (a) the kinetics of anthocyanin extraction from jambolan fruits, at all agitation frequencies, occurred at exclusively decreasing rates; (b) increment in agitation frequency substantially reduces the process time. Consequently, the 150 rpm frequency should be recommended, given the great saving of extraction time; (c) differently from what has been found in the literature, in addition to the Peleg equation, other empirical models such as the Page equation can be used to describe growth curves, determine extraction rates, and predict process times for a previously stipulated concentration.

## CONFLICT OF INTEREST

On behalf of all authors, the corresponding author states that there is no conflict of interest.

## References

[fsn3730-bib-0001] Bevington, P. R. , & Robinson, D. K. (1992). Data reduction and error analysis for the physical sciences, 2nd ed Boston, MA: WCB/McGraw‐Hill.

[fsn3730-bib-0002] Bonfigli, M. , Godoy, E. , Reinheimer, M. A. , & Scenna, N. J. (2017). Comparison between conventional and ultrasound‐assisted techniques for extraction of anthocyanins from grape pomace. Experimental results and mathematical modeling. Journal of Food Engineering, 207(1), 56–72. 10.1016/j.jfoodeng.2017.03.011

[fsn3730-bib-0003] Bucic‐Kojic, A. , Planinic, M. , Tomas, S. , Bilic, M. , & Valic, D. (2007). Study of solid‐liquid extraction kinetics of total polyphenols from grape seeds. Journal of Food Engineering, 81(1), 236–242. 10.1016/j.jfoodeng.2006.10.027

[fsn3730-bib-0004] Cacace, J. E. , & Mazza, G. (2003). Optimization of extraction of anthocyanins from black currants with aqueous ethanol. Journal of Food Science, 68(1), 240–248. 10.1111/j.1365-2621.2003.tb14146.x

[fsn3730-bib-0005] Chethana, S. , Chetan, A. N. , & Raghavarao, K. S. M. S. (2007). Aqueous two phase extraction for purification and concentration of betalains. Journal of Food Engineering, 81(4), 679–687. 10.1016/j.jfoodeng.2006.12.021

[fsn3730-bib-0006] Cissé, M. , Bohuon, P. , Sambe, F. , Kane, C. , Sakho, M. , & Dornier, M. (2012). Aqueous extraction of anthocyanins from *Hibiscus sabdariffa*: Experimental kinetics and modeling. Journal of Food Engineering, 109(1), 16–21. 10.1016/j.jfoodeng.2011.10.012

[fsn3730-bib-0007] Da Silva, W. P. , Mata, M. E. R. M. C. , Silva, C. D. P. S. , Guedes, M. A. , & Lima, A. G. B. (2008). Comportamento da secagem de grãos de feijão macassar (Vigna unguiculata (L.) Walp.) variedade sempre‐verde, como base para a determinação da difusividade efetiva e energia de ativação. Engenharia Agrícola, 28(2), 325–333. 10.1590/S0100-69162008000200013

[fsn3730-bib-0008] Da Silva, W. P. , Silva, C. M. D. P. S. , Cavalcanti, C. G. B. , Silva, D. D. P. S. , Soares, I. B. , Oliveira, J. A. S. , & Silva, C. D. P. S. (2004). LAB Fit Curve Fitting: A software in Portuguese for treatment of experimental data. Revista Brasileira de Ensino de Física, 26(4), 419–427. 10.1590/S1806-11172004000400018

[fsn3730-bib-0009] D'Alessandro, L. G. , Dimitrov, K. , Vauchel, P. , & Nikov, I. (2013). Kinetics of ultrasound assisted extraction of anthocyanins from Aronia melanocarpa (black chokeberry). Chemical Engineering Research and Design, 92(10), 1818–1826.

[fsn3730-bib-0010] Diamante, L. M. , Ihns, R. , Savage, G. P. , & Vanhanen, L. (2010). A new mathematical model for thin layer drying of fruits. International Journal of Food Science and Technology, 45(9), 1956–1962. 10.1111/j.1365-2621.2010.02345.x

[fsn3730-bib-0011] Dyrby, M. D. , Wesergaard, N. , & Stapelfeldt, H. (2001). Light and heat sensitivity of red cabbage extract in soft drink model systems. Food Chemistry, 72(4), 431–437. 10.1016/S0308-8146(00)00251-X

[fsn3730-bib-0012] Espinoza‐Perez, J. D. , Vargas, A. , Robles‐Olvera, V. J. , Rodríguez‐Jimenes, G. C. , & Garcia‐ Alvarado, M. A. (2007). Mathematical modeling of caffeine kinetic during solid– liquid extraction of coffee beans. Journal of Food Engineering, 81(1), 72–78. 10.1016/j.jfoodeng.2006.10.011

[fsn3730-bib-0013] Francis, F. J. (1982). Analysis of anthocyanins In Markakis P. (Ed.), Anthocyanins as food colors (pp. 181–207). New York, NY: Academic Press 10.1016/B978-0-12-472550-8.50011-1

[fsn3730-bib-0014] Francis, F. J. (1989). Food colourants: Anthocyanins. Critical Reviews in Food Science and Nutrition, 28(4), 273–314. 10.1080/10408398909527503 2690857

[fsn3730-bib-0015] Garcia‐Perez, J. V. , García‐Alvarado, M. A. L. , Carce, J. A. , & Mulet, A. (2010). Extraction kinetics modeling of antioxidants from grape stalk (*Vitis vinifera var. Bobal*): Influence of drying conditions. Journal of Food Engineering, 101(1), 49–58. 10.1016/j.jfoodeng.2010.06.008

[fsn3730-bib-0016] Giusti, M. M. , & Wrolstad, R. E. (2003). Acylated anthocyanins from edible sources and their applications in food systems. Biochemical Engineering Journal, 14(3), 217–225. 10.1016/S1369-703X(02)00221-8

[fsn3730-bib-0017] Hasler, C. M. (2000). The changing face of functional foods. Journal American College Nutrition., 19(5 Suppl.), 499S–506S. 10.1080/07315724.2000.10718972 11022999

[fsn3730-bib-0018] Kaleta, A. , & Górnicki, K. (2010). Evaluation of drying models of apple (var. McIntosh) dried in a convective dryer. International Journal of Food Science and Technology, 45(5), 891–898. 10.1111/j.1365-2621.2010.02230.x

[fsn3730-bib-0019] Lin, C. , Xia, G. , & Liu, S. (2017). Modeling and comparison of extraction kinetics of 8 catechins, gallic acid and caffeine from representative white teas. LWT ‐ Food Science and Technology, 10.1016/j.lwt.2017.04.028

[fsn3730-bib-0020] Liu, W. , Zhang, S. , Zu, Y. , Fu, Y. , Ma, W. , Zhang, D. , … Li, X. (2010). Preliminary enrichment and separation of genistein and apigenin from extracts of pigeon pea roots by macroporous resins. Bioresource Technology, 101(12), 4667–4675. 10.1016/j.biortech.2010.01.058 20153169

[fsn3730-bib-0021] Mercali, G. D. , Tessaro, I. C. , Norena, C. P. Z. , & Marczak, L. D. F. (2010). Mass transfer kinetics during osmotic dehydration of bananas (*Musa sapientum*, shum.). International Journal of Food Science and Technology, 45(11), 2281–2289. 10.1111/j.1365-2621.2010.02418.x

[fsn3730-bib-0022] Pan, Z. , Qu, W. , Mab, H. , Atungulu, G. G. , & McHugh, T. H. M. (2011). Continuous and pulsed ultrasound‐assisted extractions of antioxidants from pomegranate peel. Ultrasonics Sonochemistry, 18(5), 1249–1257. 10.1016/j.ultsonch.2011.01.005 21317015

[fsn3730-bib-0023] Revilla, E. , Ryan, J. M. , & Martin‐Ortega, G. (1998). Comparison of several procedures used for the extraction of anthocyanins from red grapes. Journal of Agricultural and Food Chemistry, 46(11), 4592–4597. 10.1021/jf9804692

[fsn3730-bib-0024] Silva, W. P. , Silva, C. M. D. P. S. , Gama, F. J. A. , & Gomes, J. P. (2014). Mathematical models to describe thin‐layer drying and to determine drying rate of whole bananas. Journal of the Saudi Society of Agricultural Sciences, 13(1), 67–74. 10.1016/j.jssas.2013.01.003

[fsn3730-bib-0025] Silva, W.P. , Silva, C.M.D.P.S. , Sousa, J.A.R. , & Farias, V.S.O. (2013). Empirical and diffusion models to describe water transport into chickpea (*Cicer arietinum* L.). International Journal of Food Science and Technology, 48(2), 267–273. 10.1111/j.1365-2621.2012.03183.x

[fsn3730-bib-0026] Tao, Y. , Zhang, Z. , & Sun, Da‐W (2014). Experimental and modeling studies of ultrasound‐assisted release of phenolics from oak chips into model wine. Ultrasonics Sonochemistry, 21(5), 1839–1848. 10.1016/j.ultsonch.2014.03.016 24726419

[fsn3730-bib-0027] Taylor, J. R. (1997). An introduction to error analysis, 2nd ed Sausalito, California: University Science Books.

[fsn3730-bib-0028] Veigas, J. M. , Narayan, M. S. , Laxman, P. M. , & Neelwarne, B. (2007). Chemical nature, stability and bioefficacies of anthocyanins from fruit peel of Syzygium cumini. Food Chemistry, 105(2), 619–627. 10.1016/j.foodchem.2007.04.022

